# Bone Grafting Technique in Revision ACL Reconstruction: Coring Reamer and Dowel Trick

**DOI:** 10.1016/j.eats.2022.03.024

**Published:** 2022-06-21

**Authors:** Nels D. Leafblad, Travis G. Maak

**Affiliations:** Department of Orthopaedic Surgery, University of Utah, Salt Lake City, UT, USA

## Abstract

One- or two-staged bone grafting is sometimes required for tunnel malposition and/or tunnel widening in revision anterior cruciate ligament (ACL) reconstruction. The aim of this procedure is to restore the correct position of the ACL graft in the revision setting to provide a stable and functional ACL, thereby reproducing normal knee kinematics. We present a technique that allows for a cost-effective, convenient tunnel grafting of a femoral head allograft bone dowel into both femoral and tibial defects in revision ACL reconstruction.

Failure and recurrent instability after anterior cruciate ligament (ACL) reconstruction occurs in up to 10–15% of ACL reconstructions.^1^^,^^2^ Revision ACL reconstruction is frequently complicated by tunnel malposition and/or widening. Suboptimal tunnel placement has been reported to account for up to 70–80% of ACL graft failures.^3^ Even well-positioned tunnels, however, can develop widening, which presents a substantial obstacle during revision ACL reconstruction, due to bone loss and poor graft fixation.^4^ Restoring anatomical ACL tunnel position is paramount to achieving functional stability of the knee — especially in the revision ACL setting. Therefore, one- or two-stage bone grafting of tunnels is frequently needed in both cases to achieve this end with high success rates. Mitchell et al. found no significant differences in objective or subjective outcomes or failure rates between 1- and 2-stage revision ACLRs (10.3% in the 1-stage group and 6.1% in the 2-stage group).^5^ Dragoo et al. reported that one-stage allografting resulted in improvements in knee pain, function, and stability at a minimum follow-up period of 24-months.^6^

Multiple allograft and autograft options exist, as do the techniques used to perform grafting of ACL femoral or tibial tunnel defects. These graft choices include autologous reamer-irrigator-aspirator (RIA) harvested bone from the femur,^7^^,^^8^ autologous iliac crest or proximal tibia bone graft cores,^9^ allograft bone dowels,^10^^,^^11^ and synthetic dowels.^12^ Grafts can be introduced into the defects by using tamps, grafts loaded over guide wires, or other specialized devices.^13^ Any technique chosen should be reproducible, effective, and (ideally) cost efficient. With this background in mind, we present a straightforward and cost-efficient technique to introduce a bone dowel into both femoral and tibial defects, using a single femoral head allograft for revision ACL surgery.

## Surgical Technique

**Fig 1:**
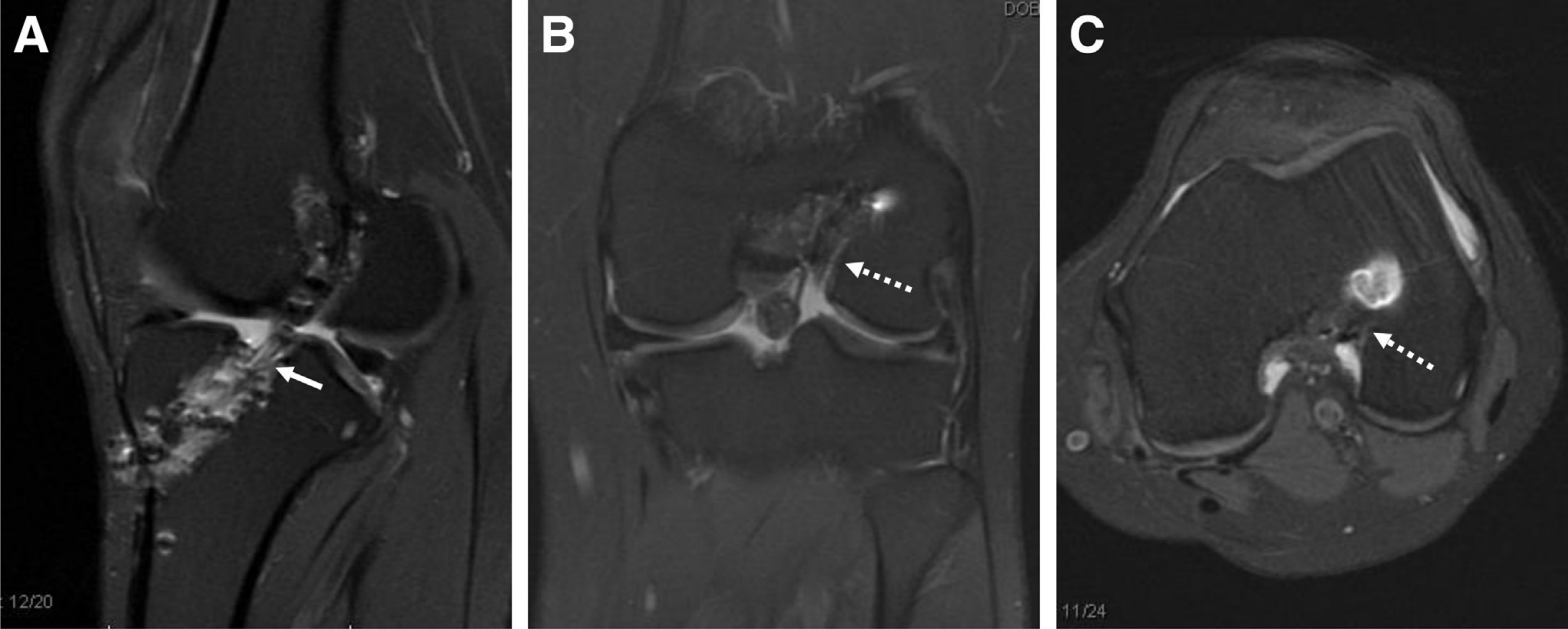
Preoperative magnetic resonance imaging (MRI) evaluation. Sagittal (A), coronal (B), and axial (C) T2-weighted MRI cuts of left knee showing a widened, posteriorly positioned tibial tunnel (solid arrow), as well as a widened and vertically malpositioned femoral socket (dashed arrows).

A thorough review of preoperative imaging is paramount for surgical planning. Fig 1 shows typical MRI images depicting malpositioned and widened tunnels in both the femur and tibia after a failed primary allograft ACL reconstruction. In this case, the femoral socket was in a vertical position, and the tibial tunnel was both widened and placed too posteriorly. (Please see the video of our technique [described below] and refer to Table 1 for the list of recommended equipment.)

**Table 1:** Equipment Required for Allograft Bone Grafting

• Standard arthroscopy equipment
• Arthroscopic shaver
• Femoral head allograft
• Cannulated reamers (Arthrex, Naples, FL)
○ Flexible reamer, low-profile reamer, or full-thickness cannulated drill
• Coring Reamer System (Arthrex, Naples, FL)
○ 7–14 mm sized coring reamer
• Pin extractor (Arthrex, Naples, FL)
• Mallet
• Powered cordless driver (Stryker, San Jose, CA)

### Patient Positioning and Diagnostic Arthroscopy

With the patient under anesthesia in a supine position, we begin with an examination to evaluate the results of the following tests: Lachman, pivot shift, dial, posterior sag/drawer, and varus/valgus stability. The operative leg is prepped and draped in the usual sterile manner. Prior to the arthroscopic portion of the procedure, we identify the prior tibial tunnel, using the previous tibial incision. A diagnostic arthroscopy is then performed using standard anterolateral and anteromedial portals to evaluate cartilage and meniscal damage, loose bodies, and the integrity of the posterior cruciate ligament. Careful evaluations of the ACL femoral and tibia tunnel apertures are also performed to determine if a one- or two-stage revision ACL is required.

If performing a one-stage revision, the surgeon must then harvest the appropriate, pre-determined graft for ACL reconstruction. We prefer to harvest ipsilateral bone patellar tendon bone (BPTB) autograft when available, as this has been shown to have lower rerupture rates in the revision setting.^14^ If performing two-stage revision, an ACL graft harvest is not performed, and attention is directed to tunnel grafting.

### Femoral and Tibial Defect Preparation

Using a shaver device, we debride the residual ACL graft and fully delineate and decorticate the femoral and tibial defects. Evaluation of the tunnel trajectory is performed using both pre-operative imaging and direct arthroscopic evaluation. Reaming and dowel grafting of the femoral tunnel should be performed in a manner similar to the original ACL to optimize dowel trajectory (typically either anteriomedial or transtibial). The width of the femoral defect to be grafted is determined, followed by sequential reaming of it with low-profile reamers. The intraosseous depth of the reamed defect is measured simultaneously during this step. The same steps are performed for the tibial tunnel. It should be reamed to the same or greater width than the femoral tunnel for later introduction of the allograft bone dowels.

### Femoral Head Allograft Harvest

**Fig 2:**
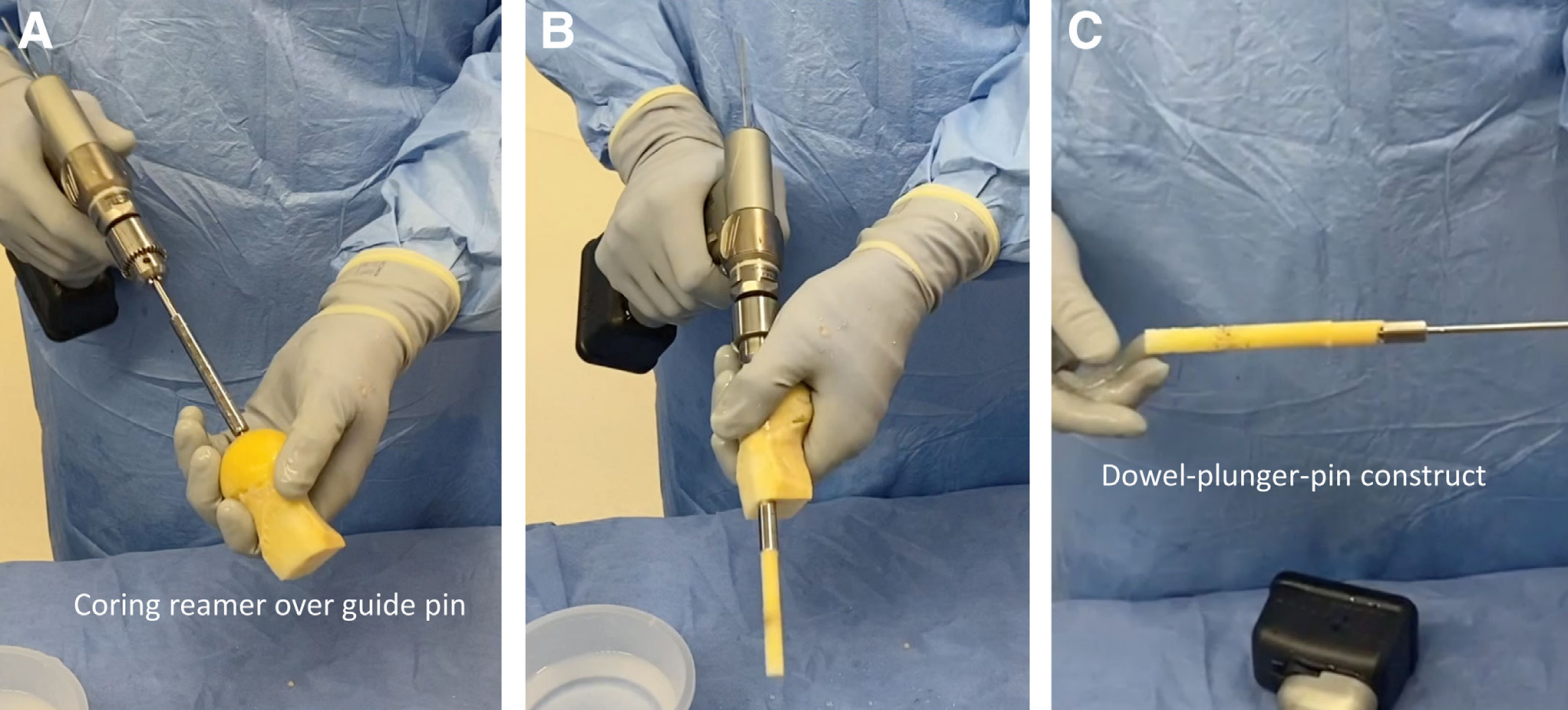
(A) A 2.4-millimeter guide pin is placed through the femoral head and neck parallel to the neck axis. (B) The coring reamer and plunger deliver the dowel over the guide pin. If needed, these steps can be repeated to create additional dowels. (C) The completed harvest shows the dowel-plunger-pin construct.

**Fig 3:**
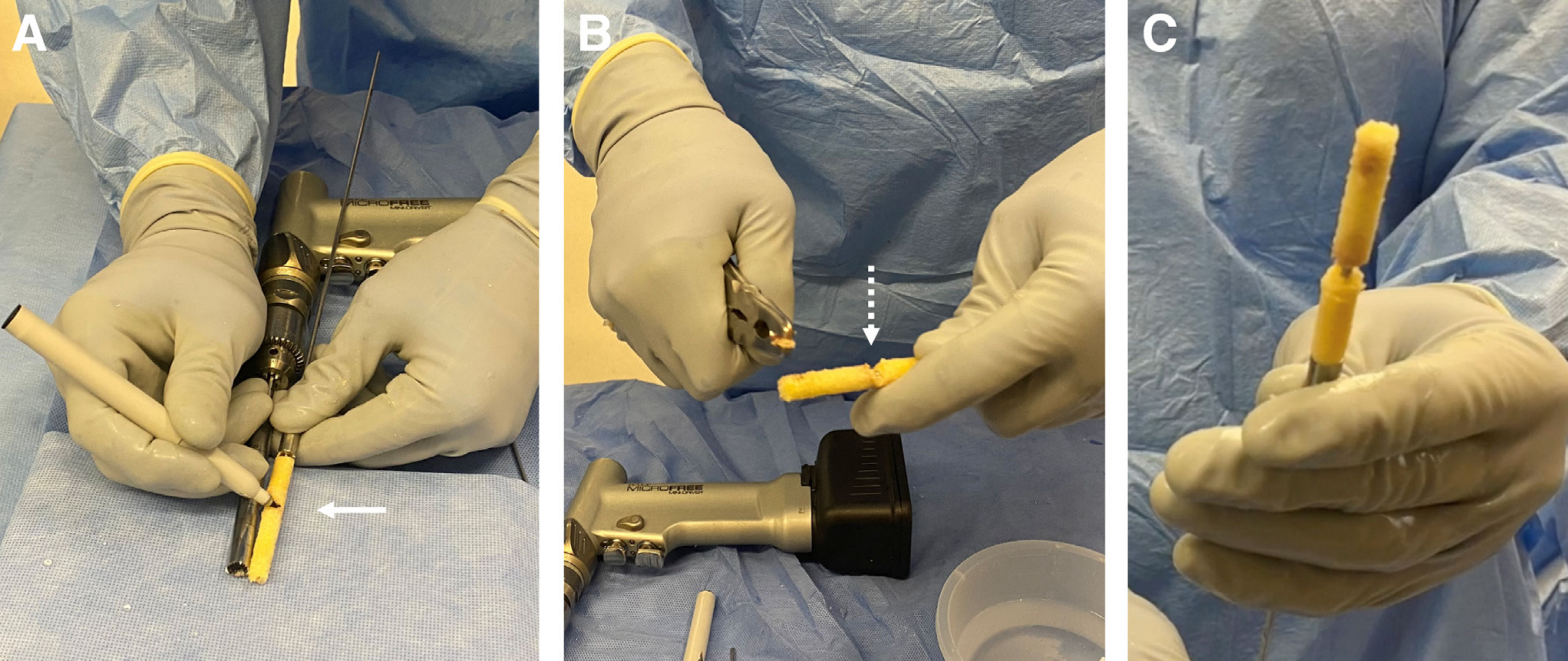
(A) Marking the femoral socket length +5 mm on the dowel (solid arrow). (B) Creating a weak spot at that mark with the use of a rongeur (dashed arrow). (C) The separated dowel fragments on the pin to be placed in the femur and tibia.

**Fig 4:**
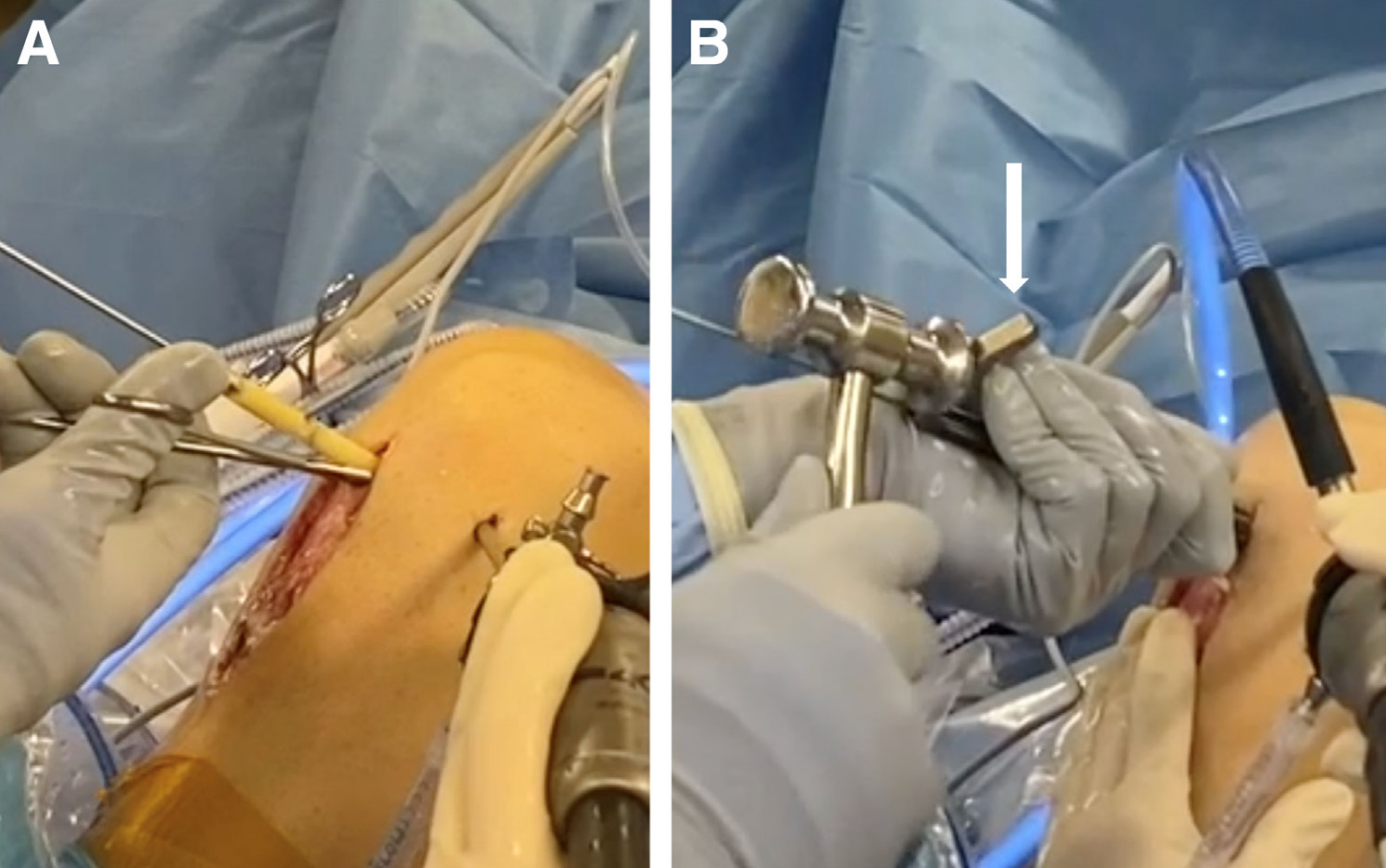
(A) The dowel-plunger-pin construct introduced into anteromedial portal. (B) Using a pin puller (white arrow) the dowel is gently tapped into position within the femoral defect.

**Fig 5:**
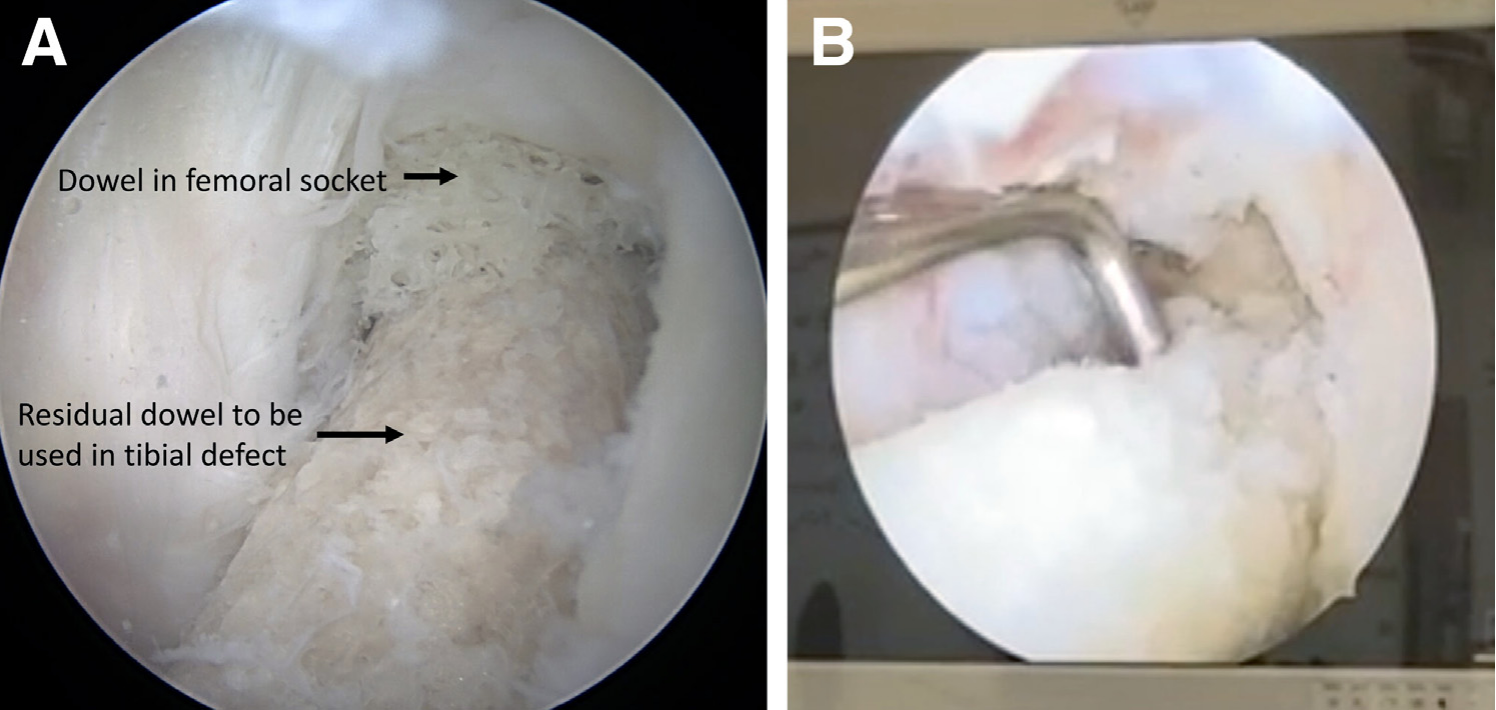
(A) Arthroscopic image of the dowel tapped into the femoral socket of a left knee. (B) A probe is used to separate the residual dowel from the femoral socket dowel.

**Fig 6:**
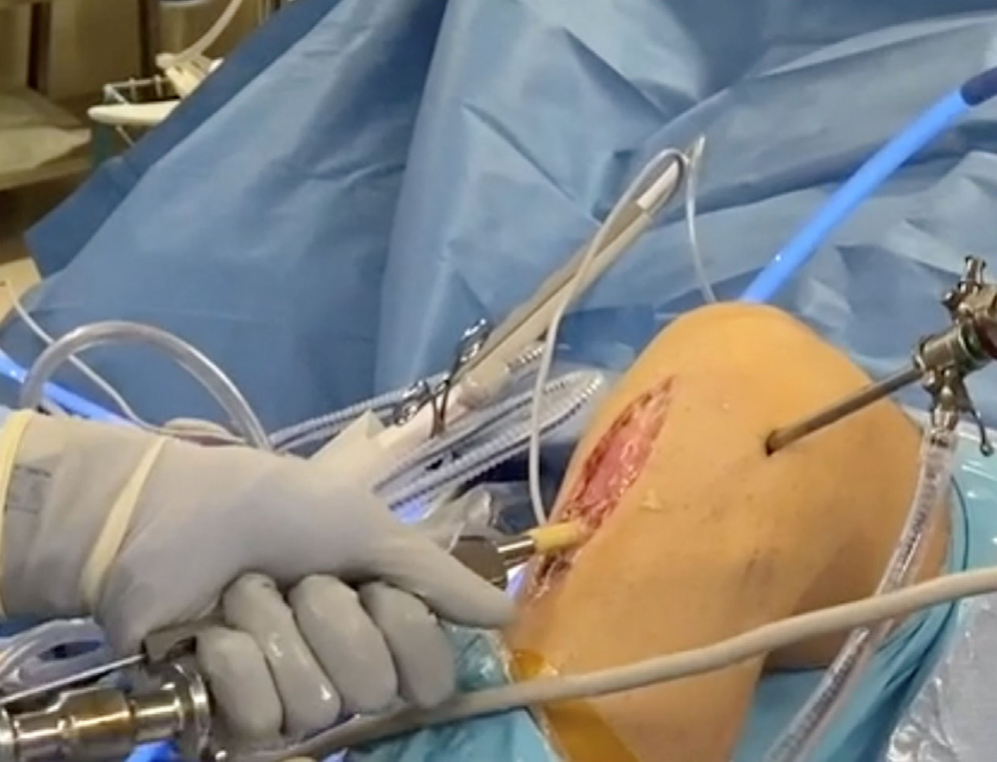
The residual dowel is tapped into the tibial tunnel/defect, again using a pin grasper/puller.

A frozen, cadaveric femoral head and neck are obtained to allow for adequate length. We then use a cannulated Coring Reamer System (Arthex, Naples, FL) to perform the harvest (Fig 2). A 2.4-mm guide pin is placed through the femoral head and neck parallel to the neck axis. A coring reamer and plunger should be used to harvest a core diameter identical to that of the reamed tunnels. Multiple cores can be obtained using a single allograft if care is taken to ensure that adequate bone is present through the neck, and if the cores are reamed in a parallel fashion. The coring reamer’s centralizing plunger with its guide pin should be left in its already cannulated position through the harvested core. This entire construct will be used for ease of grafting the associated tunnel. The allograft dowel is then marked 5 mm greater than the corresponding depth of the femoral socket, in accordance with the prior reaming depth (Fig 3). This allows for compression of the cancellous portion of the bone dowel into the tunnel. At this mark, a narrow rongeur is used to weaken the bone dowel circumferentially, leaving a small bone bridge (Fig 3). The entire construct is then introduced via the tibial tunnel or anteromedial portal, depending on the trajectory previously used to ream the femoral tunnel. The plunger-pin-dowel construct is then advanced into the femoral tunnel by gently tapping a pin puller that is solidly gripping the guide pin (Fig 4). Once the femoral dowel is fully seated and flush with the opening of the femoral defect (Fig 5), its bone bridge is then broken at the previously weakened site using a probe. The remaining dowel is removed from the knee with a powered wire driver (it will be easy to remove with the plunger-pin construct en bloc). It is then repositioned into the tibial tunnel and tapped into position as desired. If the tibial tunnel is not completely grafted, a second dowel can be harvested easily using the remainder of the first one (Fig 6).

## Discussion

There are several techniques for bone grafting tunnels in one- or two-staged ACL revision procedures with either autograft or allograft. Autogenous grafts are considered the gold standard, due to their osteoinductive, osteoconductive, and osteogenic properties. According to the systematic review by Salam et al., who evaluated graft options for tunnel augmentation, the revision ACL graft failure rates for allograft bone matrix, allograft bone chips, tibial bone autograft, and iliac crest bone autograft were 6.1%, 8.3%, 0%, and 2%, respectively (although the available data were limited and the cohort numbers quite low).^15^ These percentages indicate that there may be a slightly higher failure rate with allograft compared to autograft tunnel augmentation, but the authors acknowledge the limitations of their systematic review. Amongst these are the small number of studies included, their relatively poor level of evidence, and the heterogeneity of methods, populations, and outcome assessments, which did not allow for meta-analysis.^15^ Moreover, no structural allografts were used in this analysis. Additionally, it should be noted that multiple studies using computed tomography (CT) have reported excellent incorporation of allograft bone dowels in revision ACLR.^11^^,^^16^^,^^17^ Furthermore, harvesting autografts requires additional surgical sites and added donor site morbidity.^18^

Allograft bone tunnel augmentation has been performed with success in both one and two-stage revision ACL surgery. At 2-year follow up, Mitchell et al. found no significant differences in objective or subjective outcomes or failures between one- and two-stage revision ACLR.^5^^,^^6^ Both groups reported significant improvements in scores from pre-to post-op on the Short Form Health Survey, Western Ontario and McMaster Universities Arthritis Index, Lysholm Knee Questionnaire, and Tegner Activity Scale. Furthermore, there was no significant difference in failure rates (10.3% in the 1-stage group and 6.1% in the 2-stage group).^5^ PIoger et al. reported excellent outcomes in a single-stage revision ACL reconstruction with outside-in drilling, even in patients with tunnel widening >12 mm.^19^ Multiple authors have described their techniques for successful allograft bone tunnel augmentation.^6^^,^^19^, ^20^, ^21^

The technique we describe has multiple benefits. First, it allows for easy passage of a structural femoral head allograft bone dowel into both femoral and tibial tunnel defects. The cannulated technique with the single-dowel plunger pin construct provides a simple and reproducible arthroscopic method to allograft any tunnel size structurally. This technique can also be used in a single-stage revision reconstruction to provide structural grafting of the prior tunnel, followed by revision ACL reconstruction. Second, the femoral head allograft harvest is straightforward, is cost effective, and has no donor site morbidity. While pre-formed bone dowels are available in multiple sizes, the cost of a single pre-drilled one is often more than that of a femoral head allograft. These pre-drilled dowels may also be too short, and multiple dowels are often need for complete grafting of both tunnels, resulting in much higher costs to the patient and institution. Third, even in cases of extensive femoral and tibial tunnel widening, this allograft harvest technique allows for great flexibility in dowel diameter, which is limited only by the largest coring reamer size. The technique is also forgiving, in that if further grafting is needed, there is room to harvest multiple cores from the same femoral head allograft. Lastly, this technique is easily accomplished arthroscopically, as the allograft bone dowel can be introduced through the tibial tunnel or via anteromedial or accessory medial portals, while being controlled using the plunger/guidepin construct. Furthermore, creating a weakened bone bridge according to the defect measurements facilitates advancing a dowel into the femoral defect and then transitioning the remaining one to the tibial defect.

We have encountered no problems with this technique and foresee no major risks in using it. However, it is quite possible that aggressive malleting of the dowel may result in breakage, requiring removal of the dowel fragments and repetition or the steps. Additionally, this Coring Reamer System only allows for dowel diameters up to 13 mm. For a larger femoral socket defect, one can take a longer dowel than needed and, given judicious impaction, the graft can expand to fill the defect. For tibial defects >13 mm in diameter, the surgeon can stack two smaller dowels side by side, or fashion a custom dowel by hand. Relevant pearls and pitfalls of our technique are described in Table 2.

**Table 2:** Key Steps, Pearls, and Pitfalls of Allograft Bone Grafting

Key steps
• Conduct preoperative advanced imaging evaluation.
• Recognize concomitant pathology at time of arthroscopy.
• Determine width and depth of femoral and tibial tunnels.
• Create bone dowel from femoral head allograft using the Coring Reamer System (Arthrex, Naples, FL).
• Create weakened site in dowel at 5 mm greater than the corresponding depth of the femoral socket.
• Introduce dowel, loaded on guide wire, into femoral defect via tibial tunnel or anteromedial portal.
• Once femoral dowel is in place, use probe to break it completely at the weakened point and remove remnants.
• Reposition dowel remnants into the tibial defect.
Pearls
• Ensure you can attain the proper angle if using a trans-tibial route to introduce the dowel into the femur. If not, use an anteromedial portal or accessory medial portal.
• Measure the depth of the femoral tunnel accurately to create a dowel of the appropriate length.
• Creating a weakened site with a small bone bridge in the dowel facilitates arthroscopic breakage of it with a probe, allowing for quick transition to grafting the tibial defect.
Pitfalls
• Innacurate measuring of tunnel/socket width and depth and resultant dowel sizing error may necessitate repeat dowel harvesting.
• Aggressive malleting or introducing the dowel at an inappropriate angle risks breakage of it.

## Supplementary Data

ICMJE author disclosure forms

Video Use of the cannulated Coring Reamer System (Arthex, Naples, FL) to perform the harvest. A 2.4-mm guide pin is placed through the femoral head and neck allograft, parallel to the neck axis. A coring reamer and plunger should be used to harvest a core diameter identical to that of the reamed tunnels. The coring reamer’s centralizing plunger with its guide pin should be left in its already cannulated position through the harvested core. This entire construct will be used to easily graft the associated tunnel. The allograft dowel is then marked 5 mm greater than the corresponding depth of the femoral socket, in accordance with the prior reaming depth. This step allows for compression of the cancellous portion of the bone dowel into the tunnel. At this mark, a narrow rongeur is used to weaken the bone dowel circumferentially, leaving a small bone bridge. The entire construct is then introduced via the tibial tunnel or anteromedial portal, depending on the trajectory previously used to ream the femoral tunnel. The plunger-pin-dowel construct is then advanced into the femoral tunnel by gently tapping a pin puller that is solidly gripping the guide pin. Once the femoral dowel is fully seated and flush with the opening of the femoral defect, the dowel’s bone bridge is then broken at the previously weakened site with the use of a probe. The remaining dowel is removed from the knee using a powered wire driver. (It will come out easily with the plunger-pin construct en bloc.) The remaining dowel is then repositioned into the tibial tunnel and tapped into position as desired. Using the remainder of the first dowel, a second one can be harvested easily if the tibial tunnel is not completely grafted.
